# Core fucose identification in glycoproteomics: an ML approach addressing fucose migration in mass spectrometry

**DOI:** 10.1093/bioadv/vbaf186

**Published:** 2025-09-15

**Authors:** Yuanjie Su, Chang Jiang, Ziyue Yang, Shisheng Sun, Junying Zhang

**Affiliations:** School of Computer Science and Technology, Xidian University, Xi'an, 710071, China; School of Computer Science and Technology, Xidian University, Xi'an, 710071, China; School of Computer Science and Technology, Xidian University, Xi'an, 710071, China; Laboratory for Disease Glycoproteomics, College of Life Science, Northwest University, Xi'an, 710069, China; School of Computer Science and Technology, Xidian University, Xi'an, 710071, China

## Abstract

**Motivation:**

Core fucosylation is a common type of glycosylation that plays a significant role in biological functions. Accurate identification of core fucosylated glycopeptides is challenging due to fucose migration phenomenon during mass spectrometry. By using glycopeptides from mouse brain with FUT8 knocked out as cases and core-fucosylated high-mannose glycans in normal mouse brain as controls, the phenomena are widely observed from mass spectrometry data. The relative intensities of 10 core-related characteristic ions are used jointly as a feature vector, and a semisupervised model and a self-supervised model are developed in the feature space with robustness of the models studied.

**Results:**

Experimental results show that both models perform well, with the former superior to the latter, reaching 99.95% identification accuracy on an independent mouse brain data with FUT8 knocked out. By applying the models to wild-type mouse brain, human IgG and human serum, their dominant abundance of core fucose and/or noncore fucose are found, which is trustworthy since the effect of fucose migration is dealt with. The study highlights the great significance of trustworthy data labeling, well-defined features, and machine learning/deep learning techniques in highly reliable, accurate, and robust identification of core fucose from high-throughput mass spectrometry data.

**Availability and implementation:**

The code for core fucose identification is freely available in https://github.com/yzy-010203/core_focuse_identification.

## 1 Introduction

Core fucosylation is a common type of glycosylation that plays a significant role in the biological functions of organisms and diseases such as tumor progression, immune regulation, stem cell differentiation, fucosylation-induced physiology, and pathology (Reilly *et al.* 2019, Keeley *et al.* 2019, Xie *et al.* 2020).

For example, alterations in the core fucosylation of immunoglobulins can significantly affect their binding to Fc receptors, which is of great significance when it comes to the development of antibody drugs (Shields *et al.* 2002); core fucosylation has been shown to have an impact on protein conformation, which in turn affects properties like solubility and stability (Varki 1993); glycosylation, with core fucosylation being an essential part, plays a crucial role in guiding glycoproteins through folding, transport, and secretion processes within cells (Helenius *et al.* 2004); alterations in core fucosylation levels on epidermal growth factor receptor can lead to significant changes in downstream signaling cascades, affecting cell behaviors like proliferation and apoptosis (Miyoshi *et al.* 2006, Wang *et al.* 2020) and abnormal core fucose-modified glycoproteins in Alzheimer’s tissues suggests early detection of such changes for aiding diagnosis (Li *et al.* 2022); and viruses like influenza modify surface proteins with core fucose (Johnson *et al.* 2019). Examples are not just limited to but are much broader than the above ones.

**Figure 1.**
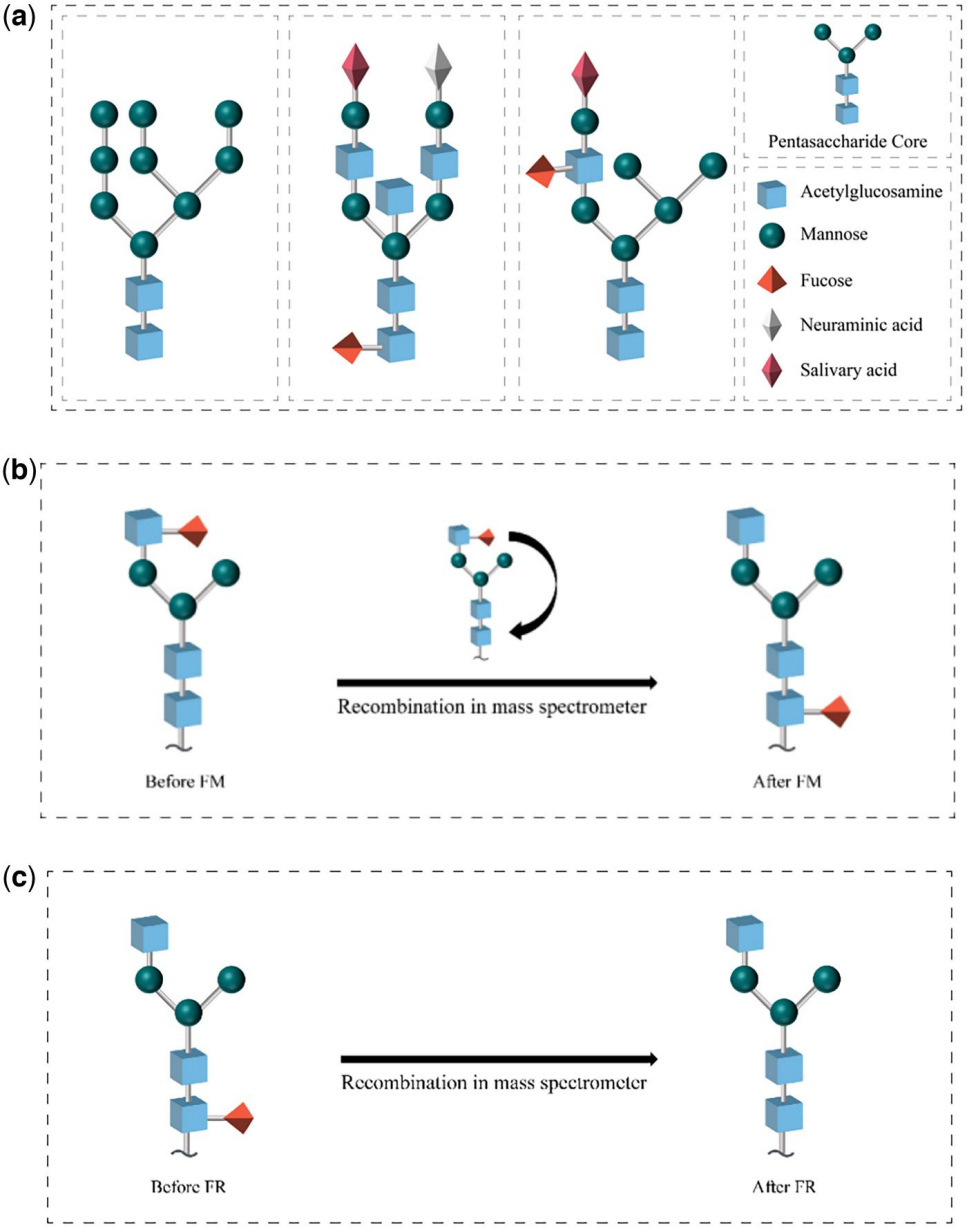
Examples of N-glycan without core-fucosylation (leftmost of A), with core fucosylation (the second left of A), with noncore fucosylation (the third left of A), and pantasaccharide core of an N-glycan (rightmost of A), and examples of N-glycan with FM (B) and with FR (C).

Pentasaccharide core is located at the base of the N-glycan and consists of two N-acetylglucosamine and three mannose monosaccharides (Lin *et al.* 2015), shown in the upper right subfigure of Fig. 1. “Core fucose” (CF) is the N-glycan with its pentasaccharide core fucosylated, particularly due to the α1,6-linkage between the fucose residue and the innermost N-acetylglucosamine (GlcNAc) of the N-glycan core; “noncore fucose” (nCF) is the N-glycan which is not a CF. Figure 1A provides N-glycan examples without fucosylation, with core fucosylation, and with noncore fucosylation, respectively. Core fucose identification (CFI) is to determine whether a glycopeptide is core fucosylated or not. The presence or absence of CF and its modification degree endow glycans with some special functions that are easily overlooked in the overall topology structure of the glycan. Compared to other parts of glycan structure, fucosylated core can serve as a relatively independent disease biomarker with higher specificity and sensitivity. This signifies the great importance of CFI.

In the past decades, mass spectrometry-based glycomics, including CFI, has made great progress and served as a popular technique for analyzing glycans as well as glycopeptides (Zeng *et al.* 2016). In mass spectrometry community, “rearrangement phenomenon” refers to the process where molecular ions undergo reconfiguration of monosaccharides or groups during fragmentation, forming new bonding relationships that did not exist in the original molecular structure and thus generating fragment ions different from those expected from normal cleavage. The phenomenon has been known for more than 25 years, regarded as a prevalent issue in the mass spectrometric analysis of protonated glycan ions that may occur in any type of mass spectrometry experiment (Mucha *et al.* 2018, Lettow *et al.* 2023).

Concerning core fucosylation, in this approach we specifically refer “fucose migration” (FM) as the phenomenon that the monosaccharide fucose on the tentacles of a noncore fucosylated N-glycan randomly attaches to the innermost GlcNAc residue of the pentasaccharide core of the N-glycan; and “fucose release” (FR) as the phenomenon of a random release of the monosaccharide fucose at the pentasaccharide core from a core fucosylated N-glycan, both being specific rearrangement relating to monosaccharide fucose (Mucha *et al.* 2018, Moge *et al.* 2023, Lettow *et al.* 2023).

FM and FR can mislead CFI: the effect of FM is that nCF is wrongly recognized to CF, since the attachment of monosaccharide fucose to the innermost GlcNAc of the N-glycan core is not due to α1,6-linkage but due to FM; and the effect of FR is that CF is wrongly recognized to nCF since the originally existent α1,6-linkage which attaches the monosaccharide fucose to the innermost GlcNAc of the N-glycan core is broken due to FR, demonstrated in Fig. 1B and C, respectively. FM and FR lead to structure confusion of CF and nCF.

The impacts exerted by FM and FR have not been seriously taken into account in the community yet. Research teams developed CFI algorithms, primarily including manual methods and now favoring more advanced machine learning-based approaches (Theodoratou *et al.* 2016, Hwang *et al.* 2020, Grabarics *et al.* 2021).

Heeyoun Hwang and his colleagues applied support vector machines and deep neural networks to CFI, achieving a commendable 99% accuracy in classifying fucosylated N-glycoproteins from human plasma (Hwang *et al.* 2020). Similarly, Theodoratou and her colleagues used support vector machines to classify glycosylation types of plasma IgG in colorectal cancer prognosis (Theodoratou *et al.* 2016). By defining and estimating a fucosylation score (F-score) from human plasma mass spectrometry data, fucosylation types of N-glycoproteins were classified with an accuracy of 99.7% (Jeong *et al.* 2020). The high accuracy of the studies indicates that machine learning is potential in CFI. However, since FM and FR were not dealt with, the data were labeled unreliably, leading to the unreliability of the model that was learned and evaluated.

Chen and his colleagues recently dealt with the problem seriously based on one feature, i.e. Y1 + Fuc/Y1 ratio (Chen *et al.* 2022). The method, referred to as Y1F/Y1 method here, successfully provides a threshold of the Y1F/Y1 ratio for CFI. The approach is suitable for spectra with collision energy of 20% or lower.

The exact mechanism of rearrangement remain elusive (Lettow *et al.* 2023). It is rather complex from the analysis in Campos *et al.* (2023). The elusive rearrangement indicates that no prior knowledge can be used, motivating the adoption of AI techniques as a unique tool for CFI.

This approach is the first one in both dealing with the effect of FM and FR and applying AI-based techniques for highly reliable CFI from the spectra in broader collision energy condition (beyond the conditions of Chens’ method in Chen *et al.* 2022). We fully take into account the impacts of FM and FR through a series of innovative designs and AI algorithms to handle the spectra conditions.

Specifically, we collect mass spectra of normal mouse brain (untagged data) and those with FUT8 gene knocked out (tagged data reliably labeled as nCF); propose 10 characteristic ions (CIs), and statistically analyze their relative intensities as features; apply AI-based techniques (semisupervised learning and self-supervised learning) in the feature space to construct highly reliable CFI models; and then compare CFI accuracy and robustness to missing CI peaks among the constructed models over two different feature normalization methods. Verification of the constructed models were conducted on an independent mouse brain data with FUT8 knocked out (FUT8-KO) reaching commendable 99.95% nCF identification accuracy. By applying the models to standard human IgG, human serum and wild-type mouse brain, dominant abundance of CF and/or nCF was obtained, which is trustworthy in the sense that the effect of FM and FR was seriously dealt with and effectively avoided. Facilitating broader condition of collision energy, the model highlights its indispensability and superiority in the relevant research field.

## 2 Methods

Methodology is studied for CFI, including collecting data, posing features, and training AI-based models, as well as proposing a method for labeling test spectra reliably, such that the performance of the trained model can be reliably evaluated.

### 2.1 Datasets

The data includes two mass spectrometry datasets for constructing CFI models, an independent spectrometry data for verification of the constructed models, and the spectrometry data from human IgG, human serum, and wild-type mouse brain as application examples of the constructed models for getting their dominant (CF or nCF) abundance.

The two datasets for constructing CFI models are the spectra of mouse brain with FUT8 knocked out and those of wild-type mouse brain from the ProteomeXchange Consortium (http://proteomecentral.proteomexchange.org) via the PRIDE partner repository with dataset identifier PXD062880. The datasets are intact glycopeptide data generated by LC-MS analyses with HCD = 33% and 20% on the Orbitrap Fusion Lumos mass spectrometer.

We select the spectra in which at least 3 out of 10 characteristic ions (see Section 2) exist for constructing CFI models. Then we get tagged dataset and untagged dataset.

The tagged dataset encompasses 23 809 mass spectra-derived from mouse brain tissue with FUT8 knockout, which are all reliably labeled nCFs by the substantial support from the result given in Yang et al. (2021): core-fucosylated glycan almost disappeared as a result of the knockout of FUT8 gene. This significantly reinforces the credibility and reliability of this approach in precisely labeling the FUT8 knock-out mass spectra as nCF.

In contrast, though endoglycosidase digestion (EndoH and EdoF) can also cleave N-linked glycoprotein, the direct action sites generally do not involve fucose (Freeze *et al.* 2010); fucosidase functions in hydrolyzing the glycosidic bonds containing fucose; however, lacks specificity to fucose in pentasaccharide core. Therefore, both are of little help in reliably labeling mass spectra.

**Figure 2.**
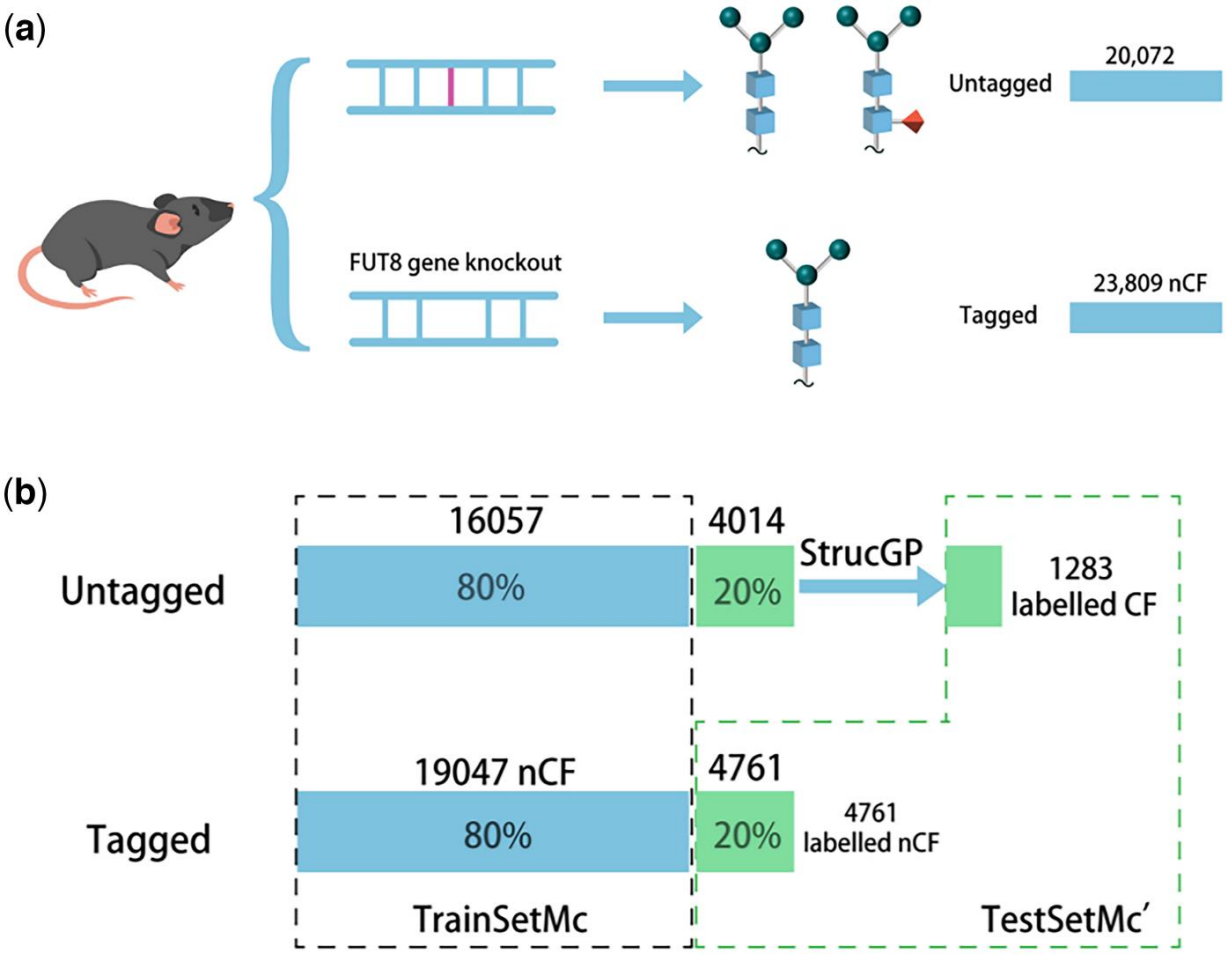
(A) Datasets for CFI modeling, and (B) training and test data for MC model from tagged and untagged datasets.

The untagged dataset includes 20 072 mass spectra from normal mouse brain tissue encompassing CF and nCF. Due to structure confusion caused by FM and FR, the dataset fails to be reliably labeled thus remains unlabeled. The two datasets, illustrated in Fig. 2A, are used for constructing CFI models.

We use the tagged and untagged dataset for constructing CFI models. The constructed models are then verified on independent spectra of mouse brain with FUT8 knocked out (FUT8-KO), and applied to the spectra of human IgG, human serum, and wild-type mouse brain, all from the ProteomeXchange Consortium with dataset identifier PXD035158, and identified by StrucGP algorithm (Shen *et al.* 2021).

### 2.2 Data usage and trustworthy labeling

Semisupervised learning and self-supervised learning are used to train CFI models, referred to as MC model and AE model, respectively.

We set different percentages of data for training MC model and for training AE model to ensure similar amount of training data. Since MC model is trained with both tagged and untagged data, while AE model is with only tagged data, the training dataset for MC model, denoted as TrainSetMC, is set to be 80% of the tagged and untagged data, with the remaining 20% as test data, denoted by TestSetMC. The training dataset for AE model, referred to as TrainSetAE, is set to be 95% of only the tagged data, with the remaining 5% tagged and untagged data as test data, referred to as TestSetAE.

TrainSetMC and TrainSetAE are used for training MC model and AE model; however, TestSetMC and TestSetAE cannot be directly used for testing the performance of MC model and AE model since some spectra in them are unlabeled. We need to reliably label them, or at least some of them, to evaluate the performance of the models reliably. In fact, only a portion of TestSetMC and a portion of TestSetAE were used. This is explained below.

Core-fucosylated mannose consists solely of mannose monosaccharides with pentasaccharide core being fucosylated.

For core-fucosylated mannose, as is well known, connecting fucose at the end of the mannose is impossible since fucose addition usually occurs in the process of glycan maturation and needs complex glycan structure as support, which the mannose lacks. This means that FM does not effect as there is no fucose present in the glycan to migrate to the pantasaccharide core of the glycan. In contrast, it is still possible that the monosaccharide fucose in the core be released by FR, making core-fucosylated mannose to noncore-fucosylated mannose. Therefore, any core-fucosylated mannose identified from mass spectrum has no effect of both FM and FR, and can always be trustworthy-labeled as “CF.” Therefore, among the untagged test data, all those which are identified to be core-fucosylated mannose can be trustworthy-labeled as “CF.”

This requires the detection of core-fucosylated mannose from mass spectra. In past few decades, mass spectrometry analysis has been used to identify glycans on glycosylation site information and glycan compositions, such as the typical Byonic 3.0 (Bern *et al.* 2012), GPQuest 2.0, pGlyco3 (Zeng *et al.* 2021), and Glyco-Decipher (Fang *et al.* 2022). Evidently, applying any such algorithms cannot provide reliable labels for test set. In contrast, StrucGP (Shen *et al.* 2021) and GlycoDeNovo (Hong *et al.* 2017) are algorithms for glycan structure prediction. Compared with the fast GlycoDeNovo, the newly developed StrucGP can identify more detailed glycan structures, the reason we chose it for the detection of core-fucosylated mannose from the untagged data in the test set. Only those identified by StrucGP software (FDR < 0.01) are labeled “CF.”

Therefore, among the spectra in TestSetMC/TestSetAE, the tagged data are trustworthy-labeled as “nCF,” and the untagged data identified by StrucGP being core-fucosylated mannose are trustworthy-labeled as “CF.” “The label of these spectra absolutely does not demonstrate the effect of FM and FR.” Using these labeled data, referred to as TestSetMC’/TestSetAE’, as the real test set for evaluating model performance, the performance is trustworthy.

The formulation of TrainSetMC and TestSetMC’ for MC model is demonstrated in Fig. 2B, with that of TrainSetAE and TestSetAE’ for AE model omitted. The related data information is summarized in Table 1.

**Table 1.** Data information and model performance.

Model	Training data	Test data	Model performance
MC model	TrainSetMC: 19 047 spectra labeled nCF, 16 057 spectra unlabeled	TestSetMC’: 4761 spectra labeled nCF, 1283 spectra labeled CF	CF Acc: 94.08% nCF Acc: **99.1%** Overall Acc: 95.32% F1 score: 0.8951
AE model	TrainSetAE: 22 618 spectra labeled nCF	TestSetAE’: 1191 spectra labeled nCF, 426 spectra labeled CF	CF Acc: **98.83%** nCF Acc: 89.86% Overall Acc: 92.16% F1 score: 0.8689

For each model, we bolded the higher value between the CF Acc and nCF Acc. This formatting choice was intended to draw attention to the superior performance metric and enable straightforward comparison.

In this approach, we select core-fucosylated mannose (identified by using StrucGP) among unlabeled data in TestSetMC and TestSetAE, and label them as CFs, for trustworthy model performance evaluation. The models facilitate CFI rather than the identification of only core-fucosylated mannose.

### 2.3 Features for CFI

**Figure 3.**
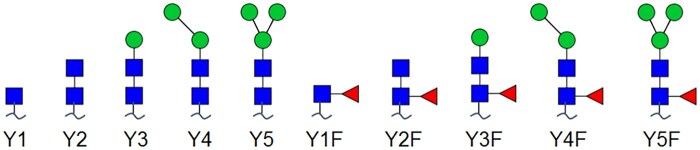
Characteristic ions for CFI (being CI1–CI10 from left to right).

We refer to the Y-ion fragments relating to pentasaccharide core as characteristic ions (CIs). These ions, denoted as Y_1_, Y_2_, Y_3_, Y_4_, Y_5_, Y_1_F, Y_2_F, Y_3_F, Y_4_F, Y_5_F, or simply CI1 to CI10, respectively, are shown in Fig. 3, where “∼” denotes peptide chain. We adopt using relative intensities of all these CIs as features, formulating a 10-dimensional feature vector ${x=[x}_{1},\;x_{2},\;x_{3},\ldots {,x}_{10}]$ for CFI. The reason is from our statistical analysis on data and our observation given in Section 3.

Two normalization methods, Max Normalization, $\;X_{i}=\frac{x_{i}}{\underset{j=1∼10}{\text{max}}\{x_{\dot{J}}\}}$, and Sum Normalization, $\;X_{i}=\frac{x_{j}}{\sum_{j=1}^{10}x_{j}}$, were employed for comparison.

### 2.4 Model construction

Two models were trained in the normalized feature space for comparison, MC model and AE model, trained by using mapping-convergence algorithm and autoencoder-based algorithm respectively.

#### 2.4.1 Mapping-convergence algorithm

Mapping convergence (MC) algorithm includes two phases, “mapping” and “convergence,” to train a model (Yu 2005).

In the mapping phase, MC algorithm uses a weak one-class classifier as an initialized classifier *h* learned from only the positives (the tagged data), denoted by *P*, to draw an initial decision boundary. The boundary separates the untagged data into positives *P*^ and negatives *N*^. Let *N* be the initially identified set of CF in the untagged dataset.

Then the convergence phase runs iteratively using SVM to maximize the margin in order to make a progressively better approximation of CFs in the untagged data. Specifically, an SVM is learned from *P* and *N*, and used to *P*^ for separating it into positives and negatives *N*^. The positives are used as the update of *P*^, and the negatives *N*^ is added to *N*. The process proceeds till convergence of *N*^ becoming vacuum. At the convergence, *N*, which is the subset of untagged data, is the sample set of identified CFs.

The training process is below:

1. Input: tagged nCF dataset *P*, untagged dataset *U*

2.  Output: CF identifier *h* and the identified CF sample set *N* in *U*

3. $\Psi_{1}$: One-Class Classifier learning algorithm, SVDD

4. $\Psi_{2}$: Binary classifier learning algorithm, SVM

5.  Using *P* and $\Psi_{1}$ to train a classifier *h*_0_

6. Classifying *U* with *h*_0_ to obtain negatives *N*_0_ and positives *P*_0_

7. Set *N*← *N*_0_, *i*← 0

Do loop

   8.1. Using *P*, *N* and $\Psi_{2}$ to train a classifier *h_i_*_+1_

   8.2. Using *h_i_*_+1_ to classify *P_i_*:

   *N_i_*_+1_←negative samples identified by *h_i_*_+1_

   *P_i_*_+1_← positive samples identified by *h_i_*_+1_

   *N*← *N* ∪*N_i_*_+1_, *i*← *i* + 1

   8.3. Repeat until $N_{i}=\emptyset$

9. return *h* = *h_i_* and *N*

#### 2.4.2 Autoencoder-based algorithm

The AE model is set be a seven-layer feedforward neural network, whose *i*th layer is with Ni neurons, where *N_i_* is set be 10, 9, 8, 7, 8, 9, 10 for *i* = 1–7. Both the input and output layer contain 10 neurons, each corresponding to the normalized relative intensity of a CI. Activation function was set be tanh, and Adam algorithm was used for the training.

After the training, AE model learns two functions: an encoding function *f* that transforms the input data to a compact representation, and a decoding function g that reconstructs the input data from the encoded representation (Zeno *et al.* 2019). We make a decision based on whether the normalized relative intensities of the CIs are well reconstructed by the model. If so, nCF is identified; otherwise, CF is identified. The algorithm is below:

1. Train an autoencoder f·g from the (untagged) dataset V;
2. Calculate the reconstruction error $e_{v}^{i}\;$of $v^{\left(i\right)}$ in dataset $V:\;e_{v}^{i}=\|v^{\left(i\right)}-g(f(v^{\left(i\right)}))\|$, and determine the threshold  α=μ+kσ, where μis the mean, σ is the standard deviation of the reconstruction errors over all the untagged data, andk is a user-parameter.
3. Calculate reconstruction error of a test mass spectrum *u*: $e_{u}^{i}=\|u-g(f(u))\|$, the spectrum is identified as CF if $e_{u}^{i}>\alpha$, and as nCF otherwise.

The user parameter k is set by trial and error in our experiment for the best model performance on TestSetAE’. The optimal setting of k is found being 0.4.

## 3 Results

Referring the spectra with FUT8 knocked out as cases, and those without knocking out FUT8 as controls, we first statistically analyze the validity of using relative intensity of the 10 CIs as features for CFI. Based on the features and trained MC model and AE model in the feature space, CFI accuracy and robustness are evaluated and compared. Then the models are validated on an independent mouse brain data with FUT8 knocked out and successfully applied to the spectra of human IgG, human serum, and wild-type mouse brain for their dominant CF/nCF abundance study.

### 3.1 Statistical analysis on characteristic ions

Identifying CF would be extremely simple if the mass spectrum be ideal in that ion fragmentation did not exhibit any FM and FR. Since only Y*i* ions exist for true nCF, the existence of Y*i*F ions indicates that the glycan is CF, and the nonexistence of all Y*i*F ions, *i* = 1–5, indicates that the glycan is nCF. However, this is not the case in experiments due to the effect of FM and FR.

**Figure 4.**
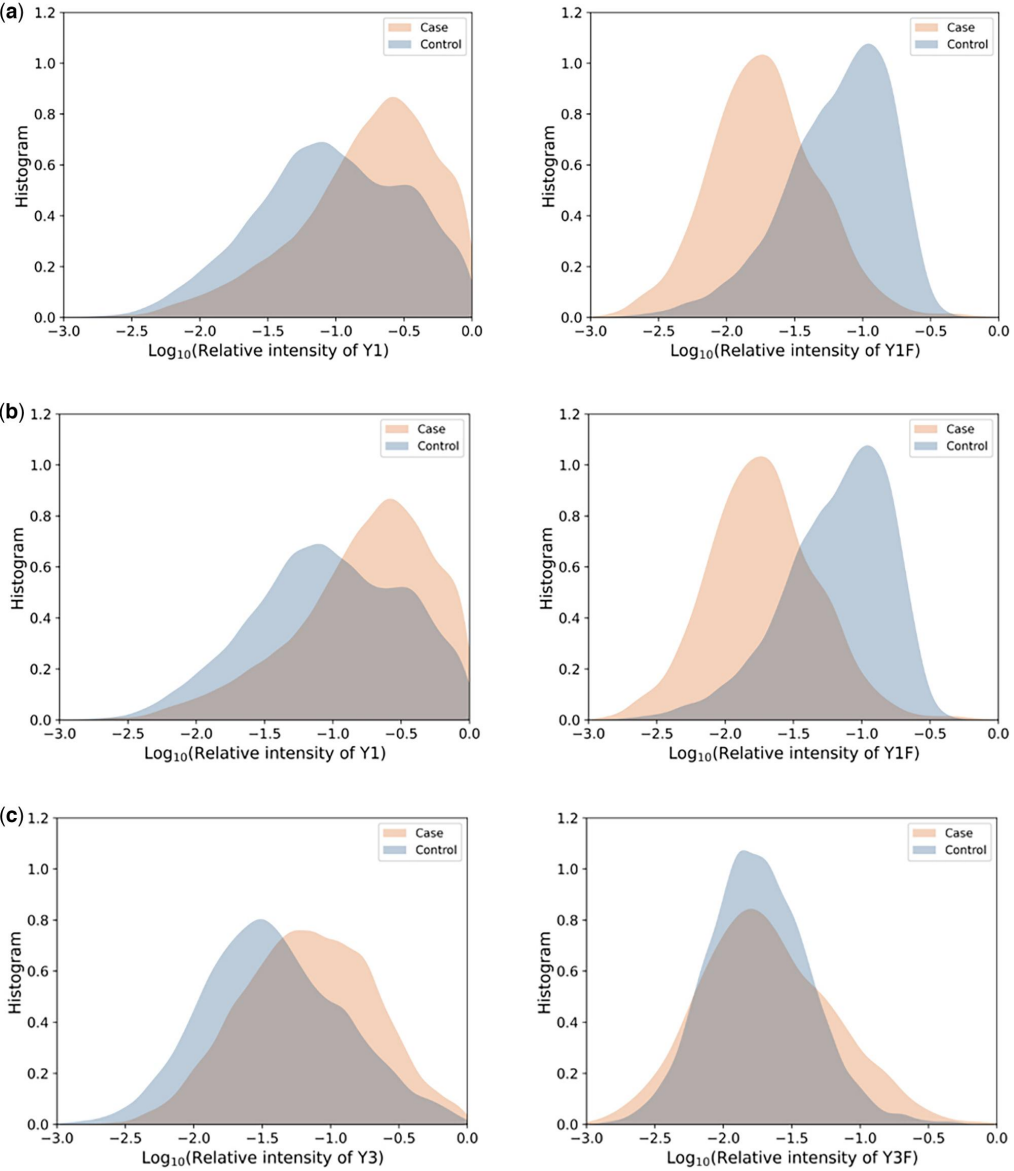
The histogram of intensity distribution of cases and controls for Y*i* ion (left column) and Y*i*F ion (right column), for A. *i* = 1, B. *i* = 2, and C. *i* = 3.

To study the effect, relative intensity of each CI is logarithmed based on 10, and the histogram/distribution of the logarithmic relative intensity for each CI for cases and for controls is drawn respectively in Fig. 4, where we omit *i* = 4 and *i* = 5 due to the limitation of the space. Figure 4 shows that the distribution for each CI is approximately Gaussian, allowing us to analyze the difference between cases and controls based on Kullback-Leibler (KL) divergence.

KL divergence $D_{\mathrm{KL}}(P||Q)$ is a non-negative and nonsymmetric measure of how one probability distribution *P* is different from another reference distribution *Q*. Here we adopt *D*(Pi,Qi)=($D_{\mathrm{KL}}(Pi||Qi)$+$D_{\mathrm{KL}}(Qi||Pi)$)/2 to represent the KL distance between the distribution of cases, *Pi*, and that of controls, *Qi*, for CI*i*, *i = *1–10. The result is shown in Table 2. Table 2 and Fig. 4 are consistent in reflecting the distribution differences between cases and controls for CI*i*, *i = *1–10.

**Table 2.** KL distance between logarithmed relative intensity distribution of cases and that of controls for characteristic ions.

CI	Distribution $N(\mu ,\sigma^{2})$		KL distance	Battle between FM and FR
	Case	Control		
Y1	(−0.78, 0.25)	(−1.04, 0.30)	0.47	FM dominates
Y2	(−1.05, 0.25)	(−1.25, 0.24)	0.34	
Y3	(−1.17, 0.23)	(−1.37, 0.23)	0.54	
Y4	(−1.04, 0.20)	(−1.37, 0.24)	1.16	
Y5	(−1.06, 0.30)	(−1.53, 0.30)	0.65	
Y1F	(−1.72, 0.16)	(−1.19, 0.16)	5.80	FR seldom dominates
Y2F	(−1.86, 0.24)	(−1.51, 0.18)	1.58	
Y3F	(−1.69, 0.24)	(−1.74, 0.14)	0.34	FR dominates
Y4F	(−1.43, 0.19)	(−1.52, 0.17)	0.17	
Y5F	(−1.35, 0.22)	(−1.53, 0.23)	0.36	

### 3.2 Observation and explanation

From Fig. 4 and/or Table 2, we have following observations:

Obs1: Differences in distribution of cases and that of controls exist in all the characteristic ions, CI*i* ions, *i = *1–10, indicating that the relative intensities of all the CIs can be used as features for the discrimination of cases and controls.

Obs2: There is a tendency for the distribution to shift from large relative intensity values in cases to small relative intensity values in controls for all Y*i*, *i = *1–5, and for Y*i*F, *i = *3–5, with the exceptions of Y1F and Y2F, observed in the corresponding subfigures in Fig. 4 and in the mean values of the distributions in Table 2, with the exceptions highlighted in bold.

Obs3: The distribution difference in Y1F and Y2F ions is the most significant among all the CIs. The intensity distribution in cases is significantly right-shifted compared to controls, with little change in variance, observed in the right subfigure of Fig. 4A and B, as well as in the KL distance bold values in Table 2. We explain the observations below.

1. The distributions are the battle result of FR and FM.
  Cases are the only nCFs thus FR does not occur on them. However, FM still occurs, leading to the presence of Y*i*F ion. This reduces the intensity of the (true) Y*i* ion in mass spectrum compared to the ideal case of without FM.
  In contrast, controls include both nCFs and CFs. For nCF, the situation is the same as analyzed above. For CF, ideally, it includes both Y*i* and Y*i*F ions. For Y*i*F ions, FR happens at random, which changes Y*i*F to Y*i*, increasing the intensity of Y*i* ions and reducing that of Y*i*F ions; for Y*i* ions, FM happens at random, which changes Y*i* ions to Y*i*F ions, reducing the intensity of Y*i* ions and increasing that of Y*i*F ions. The distribution of Y*i* and Y*i*F ion shown in Fig. 4 for controls is the outcome of the battle between FM and FR.
2. The effect of FM and FR are serious.
  The cases include only nCFs without any CFs. This means that the histograms on all the right column subfigures in Fig. 4 (golden yellow areas) should all disappear: the histograms should be all with zero values for Y*i*F, *i = *1–5. However, seen from the right column subfigures in Fig. 4, Y*i*F ions for all *i = *1–5 exist. The disappearance turns to appearance only due to the effect of FM phenomenon.
  Consider the most popular intensity of the Y*i*F with respect to that of the Y*i* for *i = *1–5. The mean of the Gaussian distribution for Y*i* and Y*i*F has been given in the second column of Table 2 for cases, from which the relative intensity is 10^mean. Then it is found that the ratio of the relative intensity of Y*i*F to that of Y*i* is 11.39%, 15.53%, 30.44%, 41.45%, and 51.53%, for *i = *1–5, respectively. This means that the impact of FM is serious in that the relative intensity of Y*i*F ideally being zeros (i.e. no appearance of Y*i*F) reaches more than 10%, and even 50% that of Y*i*, and the percentage becomes larger for larger *i*. In addition, the standard deviation from the mean for Y*i*F is 79.24% to 101.19% that for Y*i* on the logarithm of the relative intensity. Notice that Y*i*F for large *i* corresponds to large/heavy ions which can be seen from Fig. 3. All these indicate that in CIs, the more number of monosaccharides is, the more random the FM demonstrates and the more serious the impact of FM becomes. “FM phenomenon is ubiquitous, its impact is very serious and not neglectable.”
  Though it is difficult to analyze the effect of FR on distributions due to the complex battle of FM and FR, we believe that the effect of FR is also serious.
3. Effect of FR is opposite to that of FM.
  The controls include both nCFs and CFs. Seen from Table 2 is that the mean value of Y*i* ion/Y*i*F ion in controls is smaller than that in cases, *i* = 1–5/*i* = 3–5. This is explained by the stronger effect of FR than that of FM, causing more Y*i*F ions to change to Y*i* ions compared to the vice versa for *i* = 1–5, and stronger effect of FM than that of FR, causing more Y*i*F ions to change to Y*i* ions compared to the vice versa for *i* = 3–5.
  This means that for Y*i*, *i* = 1–5, FM dominates, and for Y*i*F, *i* = 3–5, FR dominates, explaining Obs2 well. This is referred to as opposite tendency of FM and FR. The tendency can also be explained simply by the fact that FM and FR have opposite effects, with the former involving the migration of a monosaccharide fucose to and the latter involving the release of the monosaccharide fucose from the pentasaccharide core of an N-glycan. Obs3 can also be explained by the opposite effects of FR and FM.
  Y1F and Y2F ions are exceptions of the tendencies in Obs2 and Obs3. Under the influence of both FM on nCF and FR on CF, the mean value of the intensity distribution of Y1F ion and that of Y2F ion for controls are greatly larger than those for cases, which is seen from the bold rows in Table 2. The KL distance of Y1F ion even exceeds 5, and that of Y2F ion even reaches the value of larger than 1.5. This indicates that for Y1F and Y2F, the dominant FR happens much less, or even seldom, than for all the other Y*i*F, *i* = 3–5. The infrequent occurrence of FR in Y1F and Y2F partially accounts for Obs4. The KL distances for Y1F and Y2F are the two largest among all distances, indicating that the relative intensities of Y1F and Y2F are the most distinguishing features between cases and controls. The relative intensities of the other CIs are also discriminant, but to a lesser extent, as shown in the KL distance column of Table 2.
  We include important battle information in the last column of Table 2, and conclude that relative intensities of CIs are reasonable features for CFI. In addition, CIs with minor domination (i.e. Y1F and Y2F) appear more significant in discriminating cases and controls.

### 3.3 Identification accuracy and robustness

MC model was trained from the dataset of TrainSetMC and tested on TestSetMC’, getting the CF accuracy of 94.08%, nCF accuracy of 99.1%, overall accuracy of 95.32%, and F1 score of 0.8951; AE model was trained from TrainSetAE and tested on TestSetAE’, getting the CF accuracy of 98.83%, nCF accuracy of 89.86%, overall accuracy of 92.16%, and F1 score of 0.8689, both summarized in the last column of Table 1. The MC model performs better than the AE model due to its higher F1 score, partly caused by the smaller size of TrainSetAE than that of TrainSetMC.

Robustness of the two models to missing CI peaks were studied. A CI peak is said missing if no spectral peak exists within a certain tolerated mass error of 20 ppm to the CI peak.

**Figure 5.**
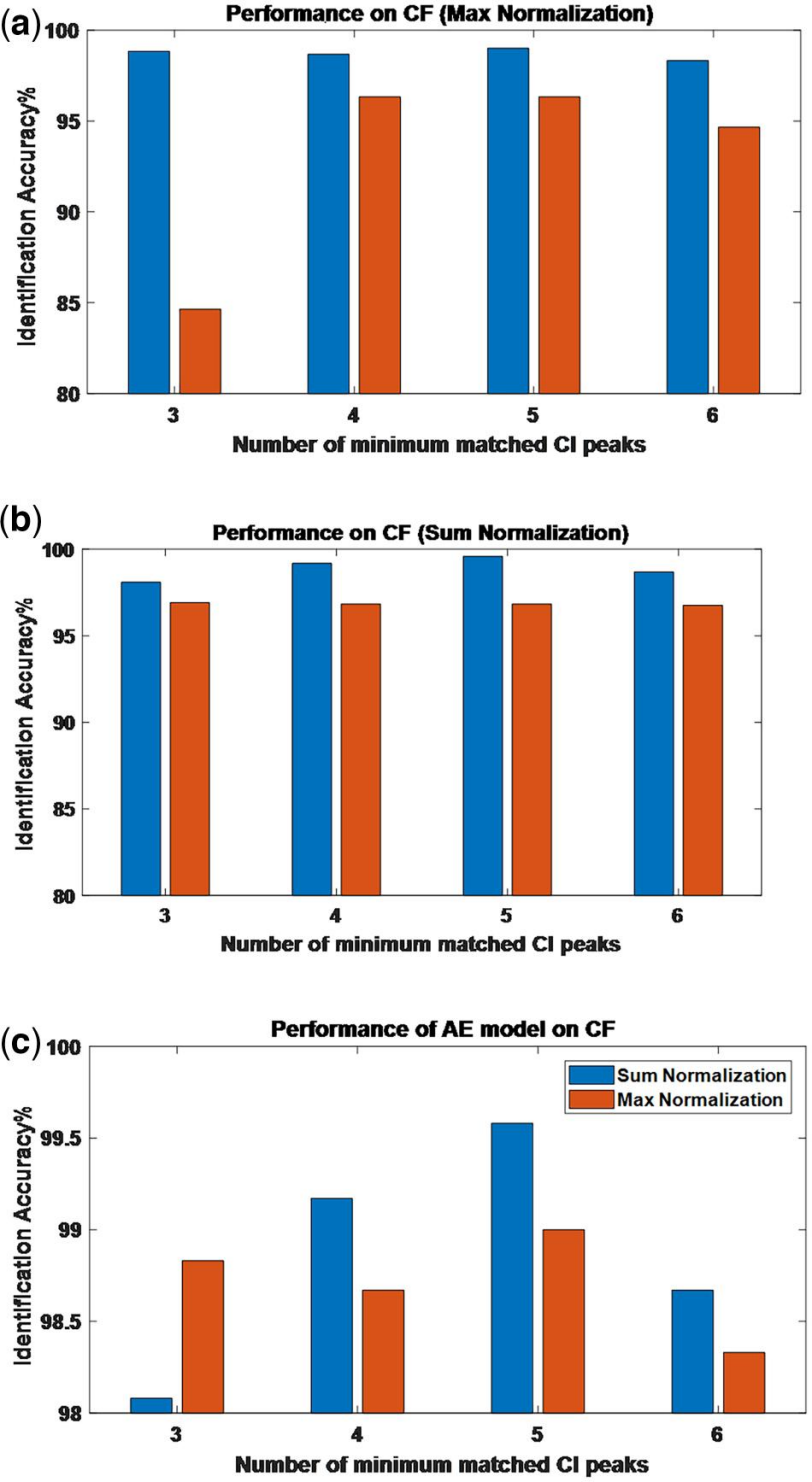
CFI performance with respect to the minimum number of matched CI peaks for AE model (left bar) and MC model (right bar) in max normalization case (A), and sum normalization case (B), and that of AE model in sum normalization (left bar) and in max normalization (right bar) case (C).

We divided training set into four (overlapped) subsets: the spectra with at least 3, 4, 5, and 6 Cis, respectively. Each of the subsets was used to retrain the two models, and the accuracy of the trained models was evaluated on TestSetMC’ and TestSetAE’, respectively. Results are shown in Fig. 5 for CFI (those for nCFI are omitted due the limitation of the space), indicating that AE model is more robust to missing CI peaks in comparison with MC model, Sum Normalization outperforms Max Normalization, and interestingly, it is not true that the more matched CI peaks, the better the model performance.

### 3.4 Model verification and application

Our MC model and AE model were verified on the independent spectra of mouse brain with FUT8 knocked out. The result is shown in the first row of Table 3.

**Table 3.** Model verification on FUT8-KO data and application to the spectra of human IgG, human serum (CE = 20%), and wild-type mouse brain (CE = 33%).

Sample	Dom. abund.	StrucGP	Y1F/Y1 method	AE model	MC model
FUT8-KO	nCF Acc	97.74	99.5	94.92	99.95
Hu IgG	CF	85.4	85.7	87.09	84.64
Hu serum	nCF	75.3	74.53	64.57	76.23
WT Mu brain	nCF	67.28	74.1	71.42	74.51

The MC model provides commendable higher nCF accuracy of 99.95% than those of the AE model, Y1F/Y1 method (Chen *et al.* 2022) and StrucGP (Shen *et al.* 2021). This again reinforce the potential of AI-based approach of well-trained model and using more features (the intensities of the 10 CIs) rather than only one feature of Y1F/Y1 ratio for CFI; it also illustrates the impact of the structural confusion caused by FM and FR on the final structural identification results, provided that the structural identification algorithm does not take into account the effects of FM and FR.

Applying the models to other organ samples of mice or even other animals is feasible. In principle, a CFI model identifies if a glycopeptide is core-fucosylated or not, regardless of which tissue or organ of which animal the mass spectrum is collected from. It is unimaginable that for the same mass spectrum, the glycan is core fucosylated if it comes from mouse brain, and it is not if it comes from porcine brain. CFI model is just to predict core-fucosylation state of the glycopeptide from a mass spectrum. Therefore, the models trained in this approach can adapt to a wide range of spectra no matter where they come from.

Yet, the model trained from only mouse brains’ limited spectra may not bias the decision-making. This is just like the situation of machine learning, where more data appears better, however small amount of data, if representative, can still train a model which can generalize well. Admittedly, more representative spectra collected from over different tissues/organs of different animals will lead to more trustworthy model compared to the current ones.

We applied the models to the spectra from human IgG, human serum and wild-type mouse brain for CFI and obtained their dominant abundance of CF and/or nCF, which are shown in Table 3: the CF abundance is 84.64% for IgG, the nCF abundance is 76.23% for serum (different from that in (Jun *et al.* 2024), where StrucGP and Y1F/Y1 method are used jointly; however, FM and FR were not dealt with in StrucGP) and the nCF abundance is 74.51% for wild-type mouse brain according to the MC model which gained higher F1 score than the AE model. The abundance can be more accurate if other types of glycans except core-fucosylated mannose, also be truly labeled in avoiding the effect of FM and FR such that the models can be improved.

Seen from Table 3, the performance of our models are not far from those of the other methods in HCD = 20%; however, a great difference can be seen in HCD = 33%: the nCF abundance obtained from the MC model is 74.51%; however, it is only 67.28% by StrucGP. Noticing that the MC model dealt with the effect of FM and FR seriously, while StrucGP ignored the effect, this means that the outer fucose of nCFs is largely migrated to pentasaccharide core especially in high collision energy, which misleads structure identification by StrucGP. This reinforces the necessity of mass spectrometry analysis tools dealing with rearrangement phenomenon.

## 4 Discussions

Here we study some issues in related references to see their connections to this approach.

1. Effect of rearrangement on mass spectra
  Campos and his colleagues made discoveries regarding glycan rearrangements at specific collision energies to explore the existence of false-positive signatures of specific outer antennary structures known as “Ghost” fragment oxonium ions (Campos *et al.* 2023). Though the work is different from this one in research object (oxonium ions versus Y ions), research task (outer antennary structure versus core fucose structure), glycopeptide range (five IgG glycopeptides versus broader types of glycopeptides), study methodology (synthesis-based versus AI-based approach), and feature dependentness (collision dependent thresholding versus relative intensities of CIs as a feature vector), it reinforces the great effect of the rearrangement on mass spectrometry, highlighting the great importance of this approach.
  According to the result in Campos *et al.* (2023), the minimal intensity threshold to prevent the mis-identification of structure-specific fragments relies on not only collision energy but also glycan structure, indicating that rearrangement is a complex phenomenon; constructing a universal model for CFI to adapt to all situations remains challenging.
2. Modeling versus thresholding
  We searched for, however failed to find, a tool or model in dealing with the rearrangement phenomenon for CFI, except for Chens’ work, providing a threshold 0.1 on Y1F/Y1 ratio for CFI (Chen *et al.* 2022).
  The low threshold of 0.1 is consistent in some sense to the ideal mass spectrum situation where ion fragmentation did not exhibit FM and FR: the existence of Y*i*F indicates that the glycan is CF. The threshold works only for collision energy of 20% or lower, which can be seen from Fig. 1D of their paper, and indicates that the effect of FM and FR appears more serious as collision energy increases, which is intuitively reasonable however the situation can be much more complex (see Fig. 3 in Campos *et al.* 2023).
  The great distinction is that we used relative intensities of ten core-related CIs as joint features (a feature vector) while Chens’ approach used only one feature, the Y1F/Y1 ratio; we train models with AI-based learning algorithms, while Chens’ approach uses experimental thresholding method. In the collision energy of 20% or lower, where the effect of FM and FR can be neglected, the feature of Y1F/Y1 ratio is a good choice, while for higher collision energy where the effect of FM and FR becomes more serious, more features and more complex AI model are a good choice.
3. CFI versus spectra prediction
  Yang and his colleagues recently introduced a deep neural network architecture able to predict mass spectra of intact glycopeptides (Yang *et al.* 2024). The two works are different in task and opposite in data flow direction: they construct a model for predicting mass spectra from glycopeptide structures to compensate the incompleteness of library coverage of spectra database, while this approach constructs a model to identify from mass spectra whether a glycopeptide is core-fucosylated or not, though both relate to mass spectra and apply AI-based technique.
4. Validation on CFI result
  In this approach, we adopt the conventional and proven effective practice in AI region of splitting data into disjoint training and nontraining set for training and evaluating a model. The only distinction lies in the fact that only a portion of the nontraining data is truly utilized as test data due to the challenge of accurately labeling all the nontraining data which is caused by the effect of FM and FR. Labeling the test spectra of core-fucosylated mannose glycans as “CF” is trustworthy, however the approach is reluctant yet due to the lack of ground truth label and no advanced alternative method.
  As long as mass spectrometry technique is used for study, the complex rearrangement phenomenon is serious and should be dealt with especially for the spectrometry data in high collision energy. However, to our knowledge, almost all the glycoprotein analysis tools to identify monosaccharide composition (Bern *et al.* 2012, Toghi Eshghi *et al.* 2015, Zeng *et al.* 2021, Fang *et al.* 2022) and glycan structure (Hong *et al.* 2017, Shen *et al.* 2021) did not consider the phenomenon, leading to less reliable result for the spectra of high collision energy. Therefore, result verification presents a challenge if relying solely on mass spectrometry data. Nevertheless, the more costly NMR spectroscopy offers a viable alternative, which can provide detailed information on connectivity of atoms within a molecule (Lin *et al.* 2015), though sample preparation requires higher purity compared to mass spectrometry study.

## 5 Conclusions

Core fucosylation plays a significant role in the biological functions of organisms and diseases. CFI from mass spectrometry data faces a significant challenge due to the phenomenon of FM and FR.

Conventional methods are either applying AI based model however ignoring the effect of FM and FR, or dealing with FM and FR phenomenon but using simple thresholding (Chen *et al.* 2022). The former leads to virtually high but not reliable CF accuracy, and the latter adapts solely to very low collision energy condition.

As the first approach for CFI dealing with FM and FR and applying AI-based technique for reliable CFI from the spectra in broader collision energy condition (beyond the conditions of Chens’ method), we conclude that the effect of rearrangement is serious and cannot be ignored especially for the spectra in high collision energy.

The main contribution of the approach is manifolds: (i) we used data with trustworthy labels for testing CFI models, where the spectrometry data of mouse brain with FUT8 gene knocked out were labeled nCF, and those identified being core fucosylated mannose by StrucGP, demonstrating no effect of FM and FR, were labeled CF, both avoiding the effect of FM and FR; (ii) we presented and statistically analyzed 10 characteristic ions (CIs) and found that the effect of FM and FR is serious and cannot be ignored especially for the spectra in high collision energy; and the relative intensities of the proposed ions are a good choice to serve as features jointly for CFI; (iii) we utilized semi-supervised MC algorithm and self-supervised AE algorithm to construct MC model and AE model in the feature space and the models were verified to be with high CFI accuracy and robustness to missing CI peaks; and (iv) the models were successfully verified on FUT8-KO spectra, and applied to standard human IgG, human serum and wild-type mouse brain obtaining their dominant CF and/or nCF abundance. The approach showcases the great potential of trustworthy data labeling, effective feature analysis and machine learning techniques for high performing CFI from mass spectrometry data.

Future work will be on expanding the study to reliably label core-fucosylated other types of glycan (other than mannose) to expand model generality, refining feature analysis and exploring deep learning to improve predictive power, integrating multiomics data and/or NMR for a holistic view of core fucosylation processes, and developing user-friendly software tools for practical application of CFI models.

## Data Availability

The data underlying this article are available in the ProteomeXchange Consortium (http://proteomecentral.proteomexchange.org) via the PRIDE partner repository with dataset identifiers PXD062880 and PXD035158.
